# Towards evidence-informed action in promoting disability inclusion in Africa

**DOI:** 10.4102/ajod.v13i0.1590

**Published:** 2024-12-05

**Authors:** Michelle Botha, Callista K. Kahonde

**Affiliations:** 1 Department of Global Health, Faculty of Medicine and Health Sciences, Stellenbosch University, Cape Town, South Africa

## Introduction

African Network for Evidence to Action in Disability (AfriNEAD) is a pan-African network promoting the implementation of disability research evidence into policy and practice through, among other activities, a triennial conference. The 7th AfriNEAD Conference took place in Cape Town from 29 November 2023 to 03 December 2023. The Conference theme was: ‘Positioning AfriNEAD: Rolling out disability research in Africa’, which is particularly pertinent as the network moves towards two decades in existence. The conference also marked the retirement of long-standing chairperson and champion of AfriNEAD, and mentor to many researchers, Prof. Gubela Mji. This special issue, therefore, offers an opportunity to look back on the research contribution of AfriNEAD to disability work in Africa (see Kahonde & Mji 2024), as well as to look forward to the role that the network can play in nurturing emerging disability studies scholarship across the continent (see Botha & Ohajunwa 2024).

The many disability-related research interests and disciplinary approaches represented at this conference evidence the interdisciplinary and intersectoral significance of disability research, and disability studies itself, in Africa. This special issue offers a taste of the disability scholarship that is currently ongoing within AfriNEAD, as well as recommendations on promoting the inclusion and empowerment of people with disabilities as stakeholders across several key spheres, including education, employment, health and rehabilitation, and, crucially, research itself.

The call for submissions to this special issue was circulated to all conference presenters. Fourteen manuscripts were received, which underwent a double blind peer review, after which 12 manuscripts were accepted. We are pleased to present this special issue of the *African Journal of Disability* (AJOD) as a record of the 7th AfriNEAD Conference.

## An overview

Kahonde and Mji (2024) present the trends in research emanating from researchers who have contributed over the course of the first six conferences of AfriNEAD. This article acts as a valuable stock-taking of the foci of disability research undertaken within AfriNEAD. However, they notice the lack of South-to- South collaborations, finding that partnerships between institutions within countries, as well as with the Global North, are more prevalent. They assert the need to develop trans-national collaborations in Africa.

We then turn to two articles in the realm of inclusive education in both basic and further education. Firstly, Kawesa et al. (2024) describe their adapting and testing of tools to measure disability inclusion in primary school classrooms in Uganda. They highlight the importance of modifying existing tools in line with the cultural and philosophical context of Africa, problematising a tendency to uncritically import interventions from other contexts. Secondly, Muzite and Gasa (2024) present their work on the lived experiences of people with disabilities attending Technical and Vocational Education and Training (TVET) colleges in South Africa. They centralise the voices of students through story exercises, presenting an intersectional analysis of barriers related to disability, socio-economic status, race and gender.

Our attention then shifts to the realm of employment. Uiras et al. (2024) present their research on the challenges faced by persons with visual impairment to accessing employment in Namibia. Qualitative data presented in this article demonstrate the impact of challenges to employment on self-acceptance and psychological well-being. Collaborations among different stakeholders are identified as crucial in promoting reasonable accommodation and optimal integration of persons with visual impairment into the labour market.

Four articles then consider experiences of people with disabilities in accessing health, rehabilitation and support services. Firstly, Watermeyer (2024) adopts a critical rehabilitation studies approach to consider the ways in which rehabilitative interventions may require people with impairments to engage in self-disciplining behaviours. Using case examples, he suggests that rehabilitation’s tendency to require individuals to strive for improvement may have implications for personal psycho-emotional well-being and collective empowerment. Secondly, Nono et al. (2024) present a scoping review on the barriers and facilitators to the use of clean intermittent catheterisation in children with spina bifida. They consider these in low-resourced contexts, offering insight into the contextual specifics, which impact this essential practice for health and well-being. Thirdly, Davids and Van Staden (2024) focus on the experiences of women who are deaf in accessing gender-based violence (GBV) support services. This qualitative study asserts the need for targeted interventions strengthened through healthcare worker training. Lastly, Mugisha et al. explore the correlation between disability severity and knowledge of HIV prevention in Uganda, providing much-needed data on this under-explored area.

A further focus at the conference, cutting across the thematic areas, was the need to develop and promote participatory research where people with disabilities are positioned as knowledge-bearers. Three articles provide useful examples and reflections. Firstly, Wickenden (2024) describes the process of conducting participatory and inclusive research into educational experiences in Kenya and Nigeria. She reflects on two projects, which employed peer researchers and utilised participatory workshops with youth with disabilities and their parents. She makes a case for the possibility of research where people with disabilities are not merely participants but directly involved as researchers. Secondly, Sikapa et al. (2024) discuss on their production of accessible digital stories on inclusive practice as a means to disseminate research evidence that is understandable for communities. This formed part of a broader study exploring the digital divide in inclusive research. Thirdly, Bannink Mbazzi et al. (2024) reflect on a process of co-creating a film on educational and employment experiences with youth with disabilities as part of a research dissemination strategy in Uganda and Ghana. They illustrate how this approach may be used to involve youth with disabilities in knowledge transfer processes.

Some key recommendations emerged from the conference on strengthening disability studies researchers in Africa. The article by Botha and Ohajunwa (2024) reports on the pre-conference event ‘Towards Strengthening African Disability Researchers’. They highlight that researchers in disability studies are engaged in both a scholarly endeavour and a personal process of conscientisation, with which they need support. Existing AfriNEAD structures are identified as holding potential to support emerging researchers with mentorship, networking and funding.

## Concluding remarks

This issue, although drawing together work in a variety of spheres, speaks strongly to the need to develop philosophical approaches, practices, tools and research methods that are grounded in the specifics of African socio-cultural contexts, which remains a core mission of AfriNEAD. The imperative to continue to foster South-to-South partnerships is clear. It is exciting to see how researchers in Africa are endeavouring to decolonise research methods and approaches. This shift brings hope of contextually relevant evidence-informed action in promoting disability inclusion in Africa.
